# Infective endocarditis with anomalous origin of coronary arteries and an abnormal aortic root bulge: A case report

**DOI:** 10.3389/fcvm.2023.1036476

**Published:** 2023-03-03

**Authors:** Guoliang Yang, Xiaoyue Lai, Chunshui Liang, Weijie Fan, Wanlei Fu, Zheng Liu, Hongmei Xia

**Affiliations:** ^1^Department of Ultrasound, Xinqiao Hospital, Army Medical University, Chongqing, China; ^2^Department of Cardiac Surgery, Xinqiao Hospital, Army Medical University, Chongqing, China; ^3^Department of Radiology, Xinqiao Hospital, Army Medical University, Chongqing, China; ^4^Department of Pathology, Xinqiao Hospital, Army Medical University, Chongqing, China

**Keywords:** aortic diverticulum, infective endocarditis, echocardiography, computed tomography, anomalous origin of the coronary artery

## Abstract

**Background:**

The aortic bulge sign possibly indicates an arterial aneurysm, pseudoaneurysm, aortic dissection, or aortic diverticulum. The aortic diverticulum is a congenital abnormality of the aorta, mainly known as an aneurysmal remnant of the dorsal fourth aortic arch or ductus arteriosus. However, the diverticulum of another part of the aorta has rarely been reported.

**Case summary:**

We report a case of a 24-year-old male with a history of oral ulcer presented with recurrent hyperpyrexia and chest pain. Echocardiography and computed tomography showed the anomalous origin of the coronary arteries, aortic valve vegetations, and a bulge at the aortic root. The patient then received a Bentall procedure. The aorta and aortic valves were replaced by a valved conduit. The bulge with a normal arterial wall at the aortic root was considered to be a diverticulum. The infective endocarditis was verified as a secondary oral-derived streptococcal infection. The patient was discharged 15 days after surgery. Post-operative echocardiography had no positive findings.

**Conclusion:**

Our case report highlights the role of multimodal cardiovascular imaging for the diagnostic workup of rare disorders, such as the presence of a diverticulum in the aortic root in a patient with endocarditis and anomalous origin of the right coronary artery.

## 1. Introduction

The aortic bulge sign found on imaging possibly indicates an arterial aneurysm, pseudoaneurysm, aortic dissection, or aortic diverticulum. The aortic diverticulum is a congenital abnormality of the aorta, mainly known as an aneurysmal remnant of the dorsal fourth aortic arch or ductus arteriosus (1, 2). However, diverticulum found in other parts of the aorta has rarely been reported except for abnormal vascular pathologies in Marfan's syndrome (3). Here we present an unusual aortic root bulge with infective endocarditis of the native aortic valve associated with concomitant anomalous origin of the coronary artery.

## 2. Case presentation

A 24-year-old male patient with recurrent fever (up to 40°C), palpitation, shortness of breath, and precordial pain after activity over the past 3 weeks was admitted to our hospital. The patient had a history of working in high-altitude areas and recurrent oral ulcers before the onset. The vital signs on admission were as follows: a body temperature of 36.4°C, heart rate of 110 beats per minute, and regular, blood pressure of 118/68 mmHg. Electrocardiogram presented sinus rhythm. Auscultation was notable for a diastolic murmur on the 3rd intercostal area of the left sternal border, and a pistol shot sound in the groin. The patient had no perineal ulcer. The acupuncture test was negative.

**Figure 1:**
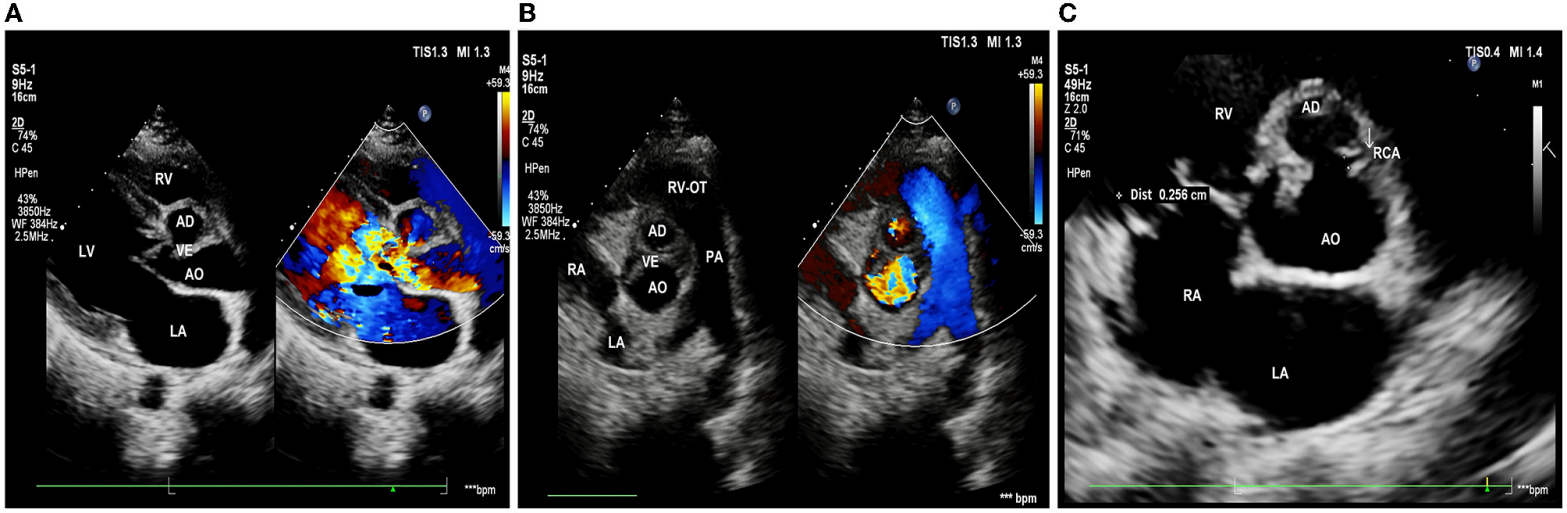
Pre-operative echocardiography. **(A)** Long-axis view of the left ventricle shows aortic valves thickening and flail motion, and color Doppler shows massive aortic regurgitation. **(B)** Short-axis view of the great arteries shows that the bulge in the aortic root communicates with the aorta, with vegetations in the aortic valve. **(C)** The right coronary artery (white arrow) is restrictive; LA, left atrium; LV, left ventricle; RV, right ventricle; AO, aorta; RVOT, right ventricular outflow tract; PA, pulmonary artery; RCA, right coronary artery; AD, aortic disease; and VE, valvular excrescence.

Echocardiography showed: left ventricle enlargement; a sonolucent area ~11.5 × 25 mm was located next to the right coronary sinus, which communicated with the dilated aortic root; a flail-like moving flocculent vegetation ~17 mm long was located in the junction area of the right and left aortic valve on the aortic side; the right and left aortic valve leaflets were both impaired, resulting in severe aortic regurgitation (Figures 1A, B); and the right coronary artery showed intramural course and was compressed by the surrounding abscess. It also showed an abnormal opening at the junction of the left and right aortic valves, with only a 2.7 mm wide ostium compared to a 4.3 mm one in the left coronary artery (Figure 1C); the ejection fraction was 69%; mild pulmonary hypertension was presented; and no obvious abnormality was observed in the aortic arch and its branches.

**Figure 2:**
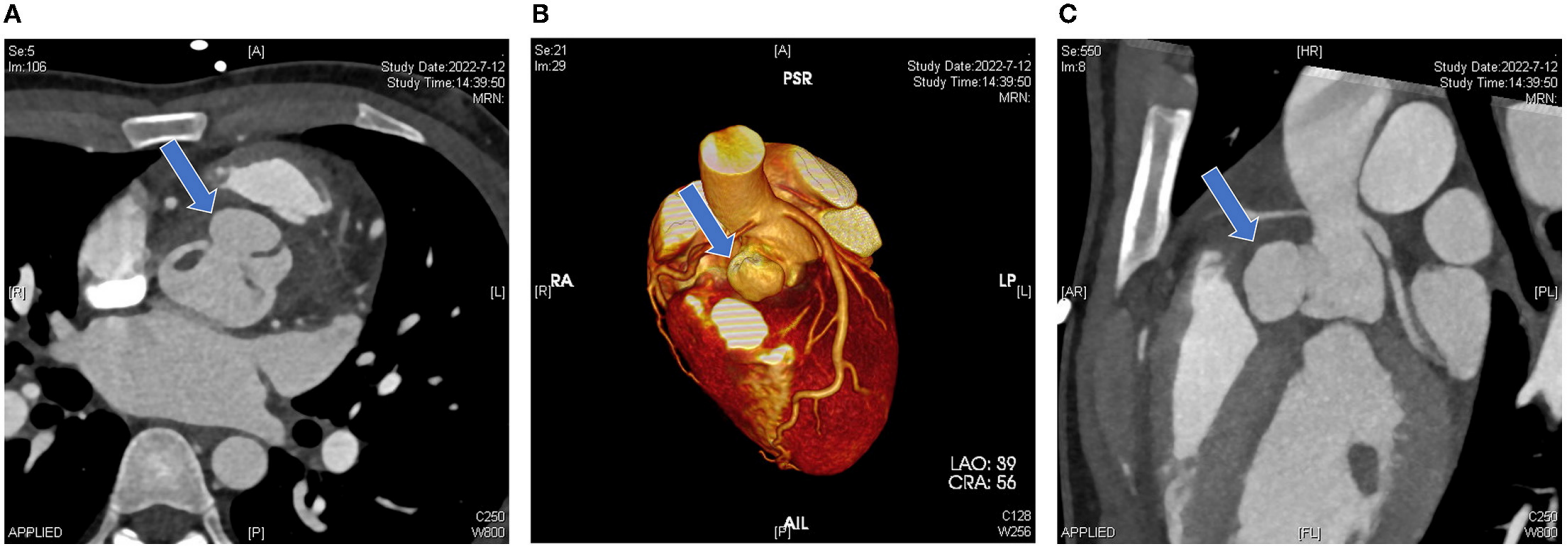
Pre-operative computed tomography. **(A)** Irregular morphology of the coronary sinuses with local anterior protrusion (blue arrow). **(B)** A three-dimensional reconstruction image shows that the left coronary artery arises from above the left coronary sinus and the right coronary artery arises from above the junction of the left and right coronary sinuses. The aortic root bulge is shown in the blue arrow. **(C)** Sagittal view showing the vertical position of the bulge (blue arrow).

Computed tomographic angiography showed that the right coronary sinus was of an irregular shape, and was pushed to the left by a 2.2 × 1.4 × 2.1 cm cystic structure in the anterior part of the aortic root. Enhancement was observed in the cystic structure, which communicated with the aorta (Figure 2A); the left coronary artery arose from above the left coronary sinus; the right coronary artery arose from above the junction of the left and right coronary sinus, and the proximal segment was surrounded by the abscess (5.1 × 2.0 cm), resulting in mild-to-moderate stenosis (Figures 2B, C). Coronary angiography was not performed. Cranial MRI indicated no intracranial infection.

Initial laboratory results were notable for leukocyte of 14.91 × 10^9^/L, albumin 28.4 g/L, procalcitonin 0.38 ng/ml, and C-reactive protein was 110.2 mg/L. Hemoglobin was 105 g/L. Cardiac troponin I (cTnI) was 0.23 pg/ml. N-terminal pro-b-type natriuretic peptide (BNP) was 609.51 pg/ml. The pre-operative blood culture result was negative. The clinical evidence suggested infective endocarditis, aortic insufficiency, aortic root abscess, and coronary heart disease.

**Figure 3:**
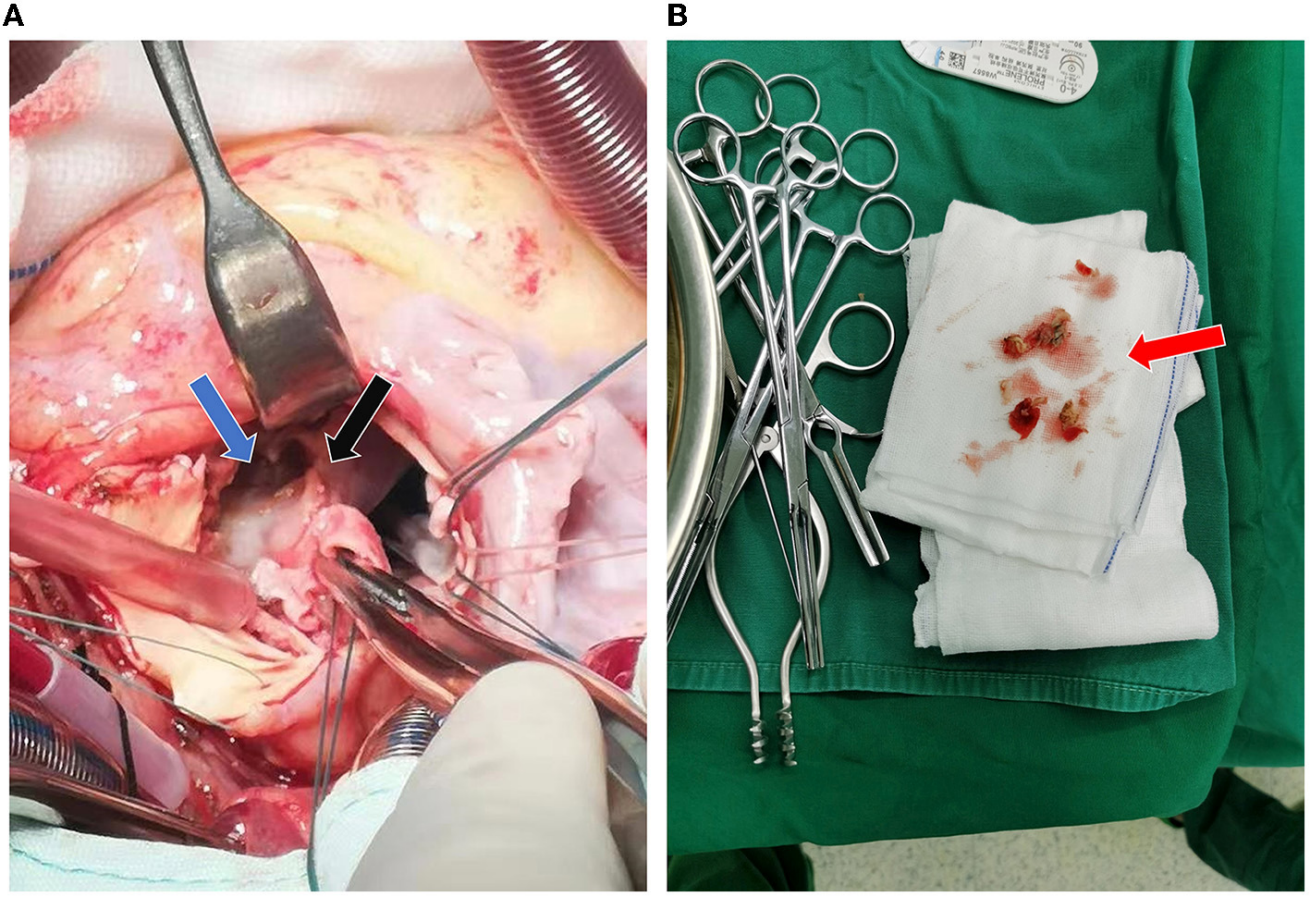
Intraoperative findings. **(A)** The aortic root bulge (blue arrow) filled with vegetation and damaged at the right coronary annulus (black arrow). **(B)** Intraoperative removal of the damaged aortic valve and part of the annulus (red arrow).

The patient then received a Bentall procedure to replace the damaged aorta and valves with a 23# valved conduit. The intraoperative findings were listed as follows: the left atrium was enlarged; there was abundant vegetation in the damaged aortic valves; and a bulge was found next to the right coronary sinus, with a complete three-layer structure (intima, media, and adventitia). The bulge communicated with the aortic root and was also full of vegetation, which compressed the right coronary sinus and blocked the right coronary ostia (Figure 3A). The left and right coronary arteries both started at the left coronary sinus and were ~4 mm apart. The right coronary artery showed ~3 mm intramural course, surrounded by purulent exudate and tissue edema. Surgical debridement was performed to clear all vegetation and remove the damaged aortic valve leaflets and part of the valvular annulus (Figure 3B). The portion of the aortic valve annulus under the right coronary sinus was reconstructed with a bovine pericardial patch. The bulge was preserved outside the conduit to have better support for the unstable annulus. A 23#SJM valved conduit was inserted, with perforation in the corresponding position of the ostia of the coronary arteries. Stenosis in the right coronary disappeared soon after the compression was removed. The coronary arteries were then anastomosed to the conduit at the assumed positions with buttons. After the surgery, the patient was sent to the intensive care unit with tracheal intubation and received intravenous ceftriaxone 2 g/day for at least 4 weeks as an anti-infective therapy.

**Figure 4:**
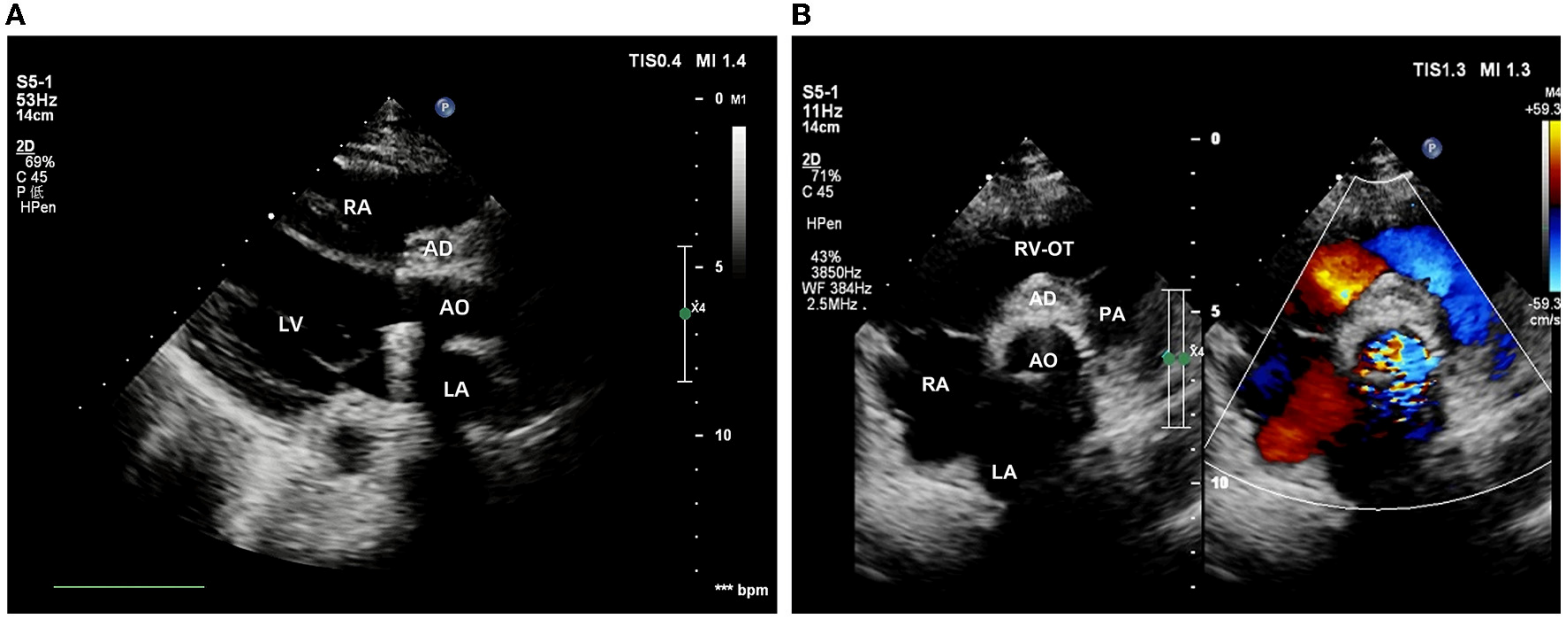
Post-operative echocardiography. **(A)** The position and function of the artificial valved conduit are normal. **(B)** The aortic root bulge is successfully closed without an internal blood flow signal.

The intraoperative and post-operative blood cultures were both negative. Therefore, we performed a post-operative high-throughput sequencing examination, which showed oral streptococcal infection. The patient then received cardiotonic therapy, anti-inflammatory treatment, and other necessary symptomatic support. Post-operative echocardiography showed that the artificial aortic conduit and valves were in normal position and functioned well (Figure 4A). The closure of the bulge was successful, with no blood flow signal inside (Figure 4B). No stenosis or obstruction was observed in the opening of the coronary arteries. Post-operative blood routine examination showed that the leukocyte returned to normal at 9.21 × 10^9^/L. The patient described that the chest pain disappeared and was discharged 15 days after the surgery. Four weeks after surgery, the follow-up echocardiography showed no positive finding.

## 3. Discussion

This is a case of an abnormal aortic root bulge, with infective endocarditis, anomalous origin of coronary arteries, and surrounding abscess. The patient was initially characterized by a recurrent oral ulcer, which was confirmed to be an oral streptococcal infection by high-throughput sequencing results. The coronary arteries originated from above the coronary sinus, together with the significantly-sized bulge compressing the right coronary sinus, leading to the local hemodynamic change. This change facilitated streptococcal colonization and vegetation and finally resulted in the formation of an aortic root abscess. The patient showed manifestations of myocardial ischemia, including chest pain, enlarged left ventricle, and abnormal BNP level. We assumed the cause was stenosis of the right coronary artery, which was the dominant coronary artery of this patient. Stenosis in the right coronary artery may result from multiple reasons: (1) anomalous origin, (2) blocked ostium under vegetations, (3) intramural course, and (4) proximal compression by the peripheral abscess.

The possible diagnosis of an aneurysm, pseudoaneurysm, and Valsalva sinus aneurysm were all considered pre-operatively, based on the morphology and location of the aneurysm-like bulge. Surgical exploration revealed its communication with the aortic root, but the bulge was a different structure from the coronary sinuses. More intriguingly, the surgical findings showed complete intima, media, and adventitia layers. Therefore, we considered the bulge to be most likely a diverticulum with the available evidence, mainly the intraoperative findings about a complete, smooth, thick, and elastic arterial wall of this structure. However, it was unfortunate in this case that we failed to perform a pathological examination because the bulge had to be preserved for safety reasons. On the one hand, the structure was necessary to support the unstable aortic annulus. On the other hand, the bulge was so big that it fell under the level of the aortic annulus, so removing the bulge might have hurt the sinoatrial node nearby.

Another possible diagnosis, of Behcet's disease in the aorta, was also considered due to the initial presentations and a history of recurrent oral ulcers. The clinical characteristics of Behcet's disease include recurrent mucosae ulcers and skin lesions. The diagnosis was rejected based on the negative result of the skin acupuncture test, which would be positive if the acupunctured skin area showed continuously expanding lesions.

The enlightenment of this interesting case is to provide a possible and necessary diagnosis consideration of diverticulum, other than pseudoaneurysm or Valsalva sinus aneurysm when an aneurysm-like structure is found at the aortic root by radiography or echocardiography. The key point of differentiating a diverticulum from a pseudoaneurysm or a Valsalva sinus aneurysm is that a diverticulum has complete intima, media, and adventitia layers structures. While the wall of a pseudoaneurysm, usually presented as a continuous interruption of the artery wall, is mainly composed of fibrous tissue, and the wall of a Valsalva sinus aneurysm is weak and thin and associated with characterized coronary sinus dilation (4, 5). The ultrasonic features of a diverticulum are very similar to normal aortic walls, while the other two diseases are different (4, 6). Therefore, distinguishing the ultrasonic features of the wall of a newfound aneurysm-like structure on echocardiography can be very important pre-operatively. However, post-operative histopathological examination is still the gold standard to confirm the diagnosis of an aortic bulge.

## 4. Conclusion

We report a case of infective endocarditis with an aortic root abscess, which was verified as a secondary oral-derived streptococcal infection in an abnormal aortic root bulge, and associated with anomalous origin of the coronary arteries and right coronary artery stenosis. The onset of the disease is rapid and life-threatening. Timely correct diagnosis and treatment are particularly important in similar cases. Due to its extremely abnormal appearance, it can be easily ignored or misdiagnosed on the first clinic visit. The enlightenment of this case is that when an aneurysm-like structure is found at the aortic root by radiography or echocardiography, the possibility of an aortic diverticulum should be considered.

## Data availability statement

The original contributions presented in the study are included in the article/Supplementary material, further inquiries can be directed to the corresponding authors.

## Ethics statement

The studies involving human participants were reviewed and approved by Xinqiao Hospital Ethics Committee. The patients/participants provided their written informed consent to participate in this study. Written informed consent was obtained for the publication of this case report.

## Author contributions

GY, CL, and HX were the main participants in the process of the case study. GY, CL, and WFu organized the data. XL and GY contributed to the manuscript. HX revised and approved the manuscript. ZL provided guidance and administrative and technical support. All authors read and approved the submitted version of the manuscript for publication.
